# Deep eutectic solvent extraction of lotus peel proanthocyanidins and its anti-senescence mechanism in t-BHP induced HepG2 cells

**DOI:** 10.3389/fnut.2026.1884044

**Published:** 2026-08-05

**Authors:** Yuling He, Yuhang Yi, Dongsheng Wang, Wenbo Wang, Runze Zhou, Jing Wen, Yanyan Li, Siyuan Liu, Tao Li, Qili Li, Chenghao Lv

**Affiliations:** 1 Laboratory of Food Function and Nutrigenomics, College of Food Science and Technology, Hunan Agricultural University, Changsha, China; 2 College of Bioscience and Biotechnology, Hunan Agricultural University, Changsha, China; 3 Hunan Provincial Key Laboratory of Liver Visceral Manifestation in Traditional Chinese Medicine, Institute of Integrative Medicine, Department of Integrated Traditional Chinese and Western Medicine, Xiangya Hospital, Central South University, Changsha, China; 4 Hunan Institute of Agricultural Product Processing and Quality Safety, Hunan Academy of Agricultural Sciences, Changsha, China

**Keywords:** anti-aging, deep eutectic solvents, *in vitro* simulated digestion, lotus peel proanthocyanidins, Nrf2/Keap1 pathway

## Abstract

Oxidative stress-induced senescence is a key driver of aging-related diseases, particularly in the liver, yet effective and safe natural interventions remain limited. This study aimed to develop a green extraction method for lotus peel proanthocyanidins (LPPs) and evaluate their antioxidant activity, monomer release during digestion, and anti-aging mechanisms. Among eight natural deep eutectic solvents (NADESs), glycerol-betaine (GL-BET, 1:1) was optimal. Using response surface methodology, the optimal extraction conditions were: water content 35%, liquid-to-solid ratio 10 mL/g, sonication time 22 min, temperature 36 °C, yielding 10.97%, which was 5.23-fold higher than 70% ethanol. During simulated digestion, six monomers were identified; proanthocyanidin B2 (PB2) showed the highest content at all stages, proanthocyanidin B3 (PB3) peaked in gastric digestion, and proanthocyanidin B4 (PB4) exhibited the strongest stability (retaining 55.32% after 4 h intestinal digestion). GL-BET, PB3, and PB4 were non-cytotoxic to HepG2 cells at 0–150 μg/mL. Pretreatment with 150 μg/mL of each compound significantly restored viability in tert-butyl hydroperoxide (t-BHP)-damaged cells, with PB4 showing the highest recovery (88.98%). PB4 reduced senescence-associated *β*-galactosidase (SA-β-gal)-positive cells from 43.23 to 15.76%, decreased reactive oxygen species (ROS) and malondialdehyde (MDA), increased superoxide dismutase (SOD), catalase (CAT), and glutathione peroxidase (GSH-Px) activities. Western blotting confirmed that PB4 upregulated nuclear factor erythroid 2-related factor 2 (Nrf2), heme oxygenase-1 (HO-1), and NAD (P)H:quinone oxidoreductase 1 (NQO1), and downregulated Kelch-like ECH-associated protein 1 (Keap1), indicating modulation of the Nrf2/Keap1/antioxidant response element (ARE) pathway. Among the three compounds tested in cellular assays (GL-BET, PB3, and PB4), PB4 exhibited the most potent anti-senescence effects. Thus, ultrasound-assisted deep eutectic solvent (DES) extraction significantly improves LPPs yield and bioactivity, providing a basis for green extraction and application of LPPs.

## 1 Introduction

Aging is an inevitable biological process accompanied by degenerative changes in multiple physiological functions (1). Oxidative stress, characterized by an imbalance between excessive ROS generation and the functional deterioration of the antioxidant defense system, plays a pivotal role in driving the aging process (2). As a vital metabolic organ, the liver is highly susceptible to oxidative damage, which can lead to hepatocyte dysfunction, aggravated inflammation, and the onset of aging-related diseases (3). Currently, clinical regimens approved by the U. S. Food and Drug Administration (FDA) for alleviating oxidative stress-induced liver injury and delaying aging-associated pathological conditions mainly include non-specific antioxidants like N-acetylcysteine, vitamin E and anti-inflammatory drugs like corticosteroids (4). However, N-acetylcysteine is primarily indicated for acute liver injury caused by acetaminophen overdose, and its long-term use may cause nausea, vomiting, and allergic reactions (5); vitamin E, despite its radical-scavenging ability, suffers from low bioavailability, and high doses have been associated with increased risks of bleeding and prostate cancer (6); corticosteroids are accompanied by severe adverse effects such as immunosuppression, osteoporosis, hyperglycemia, and hypertension, making them unsuitable for long-term use in chronic aging-related diseases (7, 8). Therefore, the identification of natural bioactive components capable of effectively reducing oxidative stress and suppressing inflammatory responses is of great significance for delaying aging and preventing associated diseases, and the exploration of safe, highly effective, and low-toxicity natural products derived from food sources has become a research hotspot (9, 10).

Lotus peel is a by-product of lotus seed processing and is rich in proanthocyanidins. Proanthocyanidins are a class of polyphenolic compounds widely distributed in the plant kingdom, formed by the polymerization of flavan-3-ol units via C-C bonds. These compounds exhibit a broad spectrum of biological activities, including antioxidant, anti-inflammatory, antitumor, and cardioprotective effects (11). However, conventional extraction methods for LPPs from low yields, leading to resource waste (12). In recent years, NADES have emerged as ideal alternatives to conventional organic solvents owing to their advantages of environmental friendliness, biodegradability, and high extraction efficiency (13). NADES are formed by combining hydrogen bond acceptors like betaine and hydrogen bond donors like glycerol, organic acids, sugars at specific molar ratios, thereby generating a unique hydrogen bond network that exhibits outstanding solubility for polyphenolic compounds (14). The successful application of NADES for proanthocyanidin extraction has been demonstrated in cottonseed hulls and Korean pine bark (15). For example, ultrasound-assisted DES extraction followed by response surface optimization achieved a yield of up to 78.58 mg/g from cottonseed hulls, with molecular dynamics simulations revealing stronger hydrogen-bonding interactions between NADES and proanthocyanidins compared with conventional solvents (16). Nevertheless, the optimization of NADES-based extraction parameters for LPPs and comparative studies with conventional solvents remain insufficiently explored. Therefore, in this study, the extraction efficiencies of 70% ethanol and eight NADESs were compared to screen the optimal solvent, and single-factor experiments followed by response surface methodology (RSM) were employed to optimize the extraction process.

Furthermore, proanthocyanidins must undergo oral, gastric, and intestinal digestive processes to be released and exert their biological activities (17, 18). *In vitro* simulated digestion models, particularly the harmonized INFOGEST protocol, can effectively evaluate the stability, release pattern, and changes in antioxidant activity of active components during digestion (19). A review highlighted that although oligomeric and polymeric proanthocyanidins largely reach the colon where they undergo microbial degradation into phenolic acids and valerolactones, slight depolymerization may also occur in the stomach and small intestine (17). A systematic investigation of the monomer composition, dynamic content changes, and bioaccessibility of LPPs during simulated gastrointestinal digestion is currently lacking. Therefore, this study employed untargeted metabolomics to comprehensively elucidate the changes in metabolites during digestion. Based on the release patterns and stability characteristics of proanthocyanidin monomers revealed by *in vitro* digestion, the anti-aging effects and molecular mechanisms of the lotus peel proanthocyanidin extract and its characteristic monomers were further investigated at the cellular level.

As a commonly used oxidative stress inducer, t-BHP significantly elevates intracellular ROS levels, triggers lipid peroxidation, and leads to mitochondrial dysfunction; consequently, it is widely employed to establish cellular oxidative stress and senescence models (20). The Nrf2/Keap1/ARE signaling pathway serves as a core regulatory system for cellular antioxidant defense (21). Published data indicate that Nrf2 activation capacity gradually declines with advancing age, and senescent cells are highly vulnerable to oxidative damage due to impaired Nrf2 signaling (22). Upon activation, this pathway upregulates downstream antioxidant enzymes such as HO-1 and NQO1, thereby enhancing cellular resistance to oxidative stress (23) Numerous proanthocyanidins, including dimers such as PB3 and PB4, have been shown to upregulate the Nrf2/ARE pathway in H₂O₂-stimulated PC12 cells (24). However, whether the lotus peel proanthocyanidin extract and its characteristic monomers PB3 and PB4 exert anti-aging effects through this pathway remains to be systematically investigated.

In this study, the optimal natural deep eutectic solvent (NADES) system for extracting proanthocyanidins from lotus peel was screened, and the release patterns and stability of proanthocyanidin monomers during simulated gastrointestinal digestion were systematically evaluated using untargeted metabolomics. In addition, the anti-aging effects of the NADES-based lotus peel proanthocyanidin (LPPs) extract and its characteristic monomers PB3 and PB4 were investigated in a t-BHP-induced HepG2 cell senescence model, with a particular focus on the Nrf2/Keap1/ARE signaling pathway.

## 2 Materials and methods

### 2.1 Materials and reagents

Lotus peel powder was sourced from Xiangtan Hongmao Xianglian Industrial Development Co., Ltd.; Catechin standard, PB3 standard, PB4 standard, and resveratrol standard were purchased from Shanghai Yuanye Bio-Technology. Anhydrous ethanol, methanol (HPLC grade), anhydrous sodium carbonate, hydrochloric acid, sodium hydroxide, ferric ammonium sulfate, 1,1-diphenyl-2-picrylhydrazyl (DPPH), and phosphate-buffered saline were obtained from Sinopharm Chemical Reagent. Rutin standard, gallic acid standard, glycerol, D-glucose, DL-lactic acid, D-sorbitol, betaine, sodium nitrite, and aluminum nitrate were purchased from Shanghai Macklin Biochemical Technology. Folin–Ciocalteu reagent was obtained from Beijing Solarbio Science & Technology. *α*-Amylase, pepsin, pancreatin, porcine gastric pepsin, trypsin, and bile salts were purchased from Sigma-Aldrich Corporation. HepG2 cells were obtained from Huazhong Agricultural University. Minimum Essential Medium (MEM) medium (Catalog No. 31985062) was purchased from Thermo Fisher Scientific (China). Fetal bovine serum (FBS) was obtained from Bio-Techne Shanghai. Bicinchoninic acid (BCA) protein assay kit was purchased from Biosharp (Canada). 5 × protein loading buffer, Cell Counting Kit-8 (CCK-8), and malondialdehyde (MDA) assay kit (Catalog No. S0131S, 100 T) were purchased from Beyotime (Shanghai). Superoxide dismutase (SOD) kit and glutathione peroxidase (GSH-Px) kit were obtained from Nanjing Jiancheng. Catalase (CAT) activity assay kit (Catalog No. Bc0205) was purchased from Beijing Solarbio Science & Technology.

### 2.2 Experimental instruments

The rotary evaporator was purchased from Shanghai Yarong Biochemical Instrument (Shanghai, China). The pH meter was obtained from Shanghai Leici Product Marketing Center (Shanghai, China). The electronic balance was supplied by Sartorius Scientific Instruments (Beijing, China). The multifunctional microplate reader (fluorescence) was purchased from Thermo Fisher Scientific (Vantaa, Finland). The constant temperature water bath was purchased from Beijing Yongguangming Medical Instrument (Beijing, China). The metal bath was obtained from Tiangen Biotech (Beijing, China). The thermometer was supplied by Beijing Liuyi Biotechnology (Beijing, China). The constant temperature air-bath shaker was purchased from Shanghai Huaxi Scientific Instrument (Shanghai, China). The −80 °C refrigerator was obtained from Thermo Fisher Scientific (Waltham, MA, USA). The vortex mixer was purchased from Shanghai Huxi Analytical Instrument Factory (Shanghai, China). The clean bench was supplied by Suzhou Purification Equipment (Suzhou, China). The high-speed refrigerated benchtop centrifuge was obtained from Hunan Xiangyi Laboratory Instrument Development (Hunan, China). The biomolecular imager was purchased from Cytiva (Uppsala, Sweden). The vertical electrophoresis apparatus was supplied by Bio-Rad Laboratories, Inc. (Hercules, CA, USA). The cell culture incubator was obtained from Thermo Fisher Scientific (Waltham, MA, USA).

### 2.3 Extraction of proanthocyanidins from lotus peel

The 70% ethanol reflux extraction was performed as described by Zhang et al. (25) with slight modifications. Briefly, 1. 00 g of lotus peel powder was extracted with 70% ethanol at a liquid-to-solid ratio of 20 mL/g under reflux conditions in an 80 °C water bath for 2 h. After centrifugation at 4000 rpm for 10 min, the supernatant was collected as the crude extract.

Eight types of natural NADES were prepared according to previously reported methods (26). Betaine was used as the hydrogen bond acceptor (HBA), while glycerol, D-sorbitol, DL-lactic acid, and D-glucose served as hydrogen bond donors (HBD). The NADES were formulated at betaine-to-HBD molar ratios of 1:1 or 1:2 as follows: NADES 1 (glycerol, 1:1), NADES 2 (glycerol, 1:2), NADES 3 (D-sorbitol, 1:1), NADES 4 (D-sorbitol, 1:2), NADES 5 (DL-lactic acid, 1:1), NADES 6 (DL-lactic acid, 1:2), NADES 7 (D-glucose, 1:1), and NADES 8 (D-glucose, 1:2). The water content in each system was adjusted to 30% (27). After heating at 80 °C with stirring until a clear homogeneous phase formed, the NADES were cooled to room temperature. Lotus peel powder (1. 00 g) was extracted with 20 mL of each NADES under the following conditions: liquid-to-solid ratio 20 mL/g, water content 30%, ultrasonic temperature 40 °C, and time 30 min. The mixture was centrifuged at 4000 rpm for 10 min, and the supernatant was used to determine the proanthocyanidin content and calculate the extraction yield. The NADES system giving the highest yield was selected as the optimal system, with 70% ethanol extraction as the control.

### 2.4 Calculation of proanthocyanidin yield

The proanthocyanidin content of the lotus peel extract was determined using the vanillin-HCl method, as previously described (28). All analyses were performed in triplicate. The yield of LPPs was calculated using Equation (1):

$$\text{The yield of LPPs}\;(\%)=\frac{C*V*n}{1000*M}*100$$ (1)

Where:

C: proanthocyanidin concentration determined from the standard curve, in micrograms per milliliter (μg/mL);

V: volume of the sample solution used in the reaction (mL);

n: dilution factor of the sample solution;

M: dry mass of the lotus peel sample (mg).

The arithmetic mean of two independent determination results obtained under repeatability conditions was calculated and retained to three significant figures. The measured result was adjusted by subtracting the blank value.

### 2.5 Single-factor experiments

After determining the optimal NADES system, single-factor experiments were conducted to evaluate the effects of water content (10, 20, 30, 40, 50%), liquid-to-solid ratio (10, 15, 20, 25, 30 mL/g), sonication time (10, 20, 30, 40, 50 min), and sonication temperature (20, 30, 40, 50, 60 °C) on the proanthocyanidin yield. Each factor was varied while keeping the other three constant at the following baseline conditions: water content 30%, liquid-to-solid ratio 20 mL/g, sonication time 30 min, and sonication temperature 40 °C.

### 2.6 Response surface experimental design

RSM was performed as described by Bezerra et al. (29). Briefly, a four-factor, three-level Box–Behnken design was used with the proanthocyanidin yield (multiplied by 100) as the response variable Y. The specific conditions are shown in Supplementary Table S1.

### 2.7 Anti-senescence activity variation during *in vitro* simulated digestion

Simulated salivary fluid (SSF), simulated gastric fluid (SGF), and simulated intestinal fluid (SIF) electrolyte stock solutions were prepared in accordance with references (30, 31). The sample solution was sequentially subjected to oral (2 min, 37 °C), gastric (1,2,4 h, 37 °C, pH 2.0), and intestinal (1,2,4 h, 37 °C, pH 7.0) digestion phases using simulated salivary, gastric, and intestinal electrolyte stock solutions with the appropriate digestive enzymes (*α*-amylase, pepsin, pancreatin, and bile salts). CaCl₂ was added directly to the final digestion mixture to avoid precipitation. The composition and proportion of the electrolyte are shown in Supplementary Table S3. The DPPH and ABTS radical scavenging activities were determined according to the method of Sridhar and Charles (32).

### 2.8 Non-targeted metabolomics analysis

To explore metabolite differences among samples, the grouping information is presented in Supplementary Table S4. Metabolite measurements were performed on a Waters Acquity UPLC system coupled with a PDA detector and an AB TripleTOF 6,600 mass spectrometer, using a Waters UPLC HSS T3 column. The mobile phase consisted of water and acetonitrile, both containing 0.1% formic acid. Samples were analyzed in positive and negative ionization modes, with quality control (QC) samples injected every 10 runs to monitor signal stability (detailed conditions in Supplementary Tables S5, S6). Raw data were processed for peak detection, alignment, and integration using standard software. Metabolite identification was confirmed by retention time, exact mass (mass error<5 ppm), and MS/MS fragmentation, against in-house and public databases. Multivariate analyses (PCA and PLS-DA) were performed. Differential metabolites were selected based on fold change thresholds, and pathway analysis was conducted using the KEGG database. For bioaccessibility assessment during the simulated intestinal digestion phase, the end point of gastric digestion (G4I0) was used as the initial reference to determine the relative content of each proanthocyanidin monomer at different intestinal digestion time points (1, 2, and 4 h). The bioaccessibility index of each monomer during the intestinal phase was calculated according to Equation 2:

$$\text{Bioaccessibility index}\;(\%)=\frac{A_{0}-A_{t}}{A_{0}}\times 100$$ (2)

Where:

A_0_ represents the relative content of the given proanthocyanidin monomer at the G4I0 stage (0 h of intestinal digestion), and A_t_ represents the relative content of the same monomer after t hours (1, 2, or 4 h) of intestinal digestion. A higher bioaccessibility index indicates that the monomer is more readily degraded, transformed, or absorbed in the intestinal environment (32).

### 2.9 Cell-based assessment of anti-aging efficacy and underlying mechanisms

HepG2 cells (obtained from Huazhong Agricultural University) were seeded in a complete MEM medium (Thermo Fisher Scientific) supplemented with 10% fetal bovine serum (FBS, Bio-Techne Shanghai) and 1% penicillin–streptomycin, and then subcultured at 37 °C in a 5% CO₂ atmosphere.

#### 2.9.1 Cell viability assay

Cell viability was determined using the CCK-8 assay (33). HepG2 cells were seeded into 96-well plates (Corning, USA) at a density of 2 × 10^4^ cells per well (100 μL per well) and allowed to attach for 24 h, then treated with various concentrations of GL-BET, PB3, PB4, or 10 μM resveratrol for another 24 h. Subsequently, 10 μL of CCK-8 solution was added to each well, and incubated for 30 min at 37 °C in the dark. Absorbance was recorded at 450 nm using a microplate reader (Thermo Fisher Scientific).

#### 2.9.2 Establishment of the t-BHP-induced cell senescence model

The t-BHP-induced HepG2 cell senescence model was established as described previously (34). HepG2 cells were seeded at a density of 2 × 10^4^ per well in a 96-well plate and cultured for 24 h. The original culture medium was then removed, and fresh MEM basal medium containing different concentrations of t-BHP (0, 5, 10, 15, 20, 25, 30 μg/mL) was added to each well (0.1 mL per well), with three replicates for each concentration. After incubation for 2 h, 10 μL of CCK-8 solution was added to each well and incubated at 37 °C in the dark for 30 min. Absorbance was measured at 450 nm. The t-BHP concentration resulting in approximately 50% cell viability was selected as the optimal concentration for subsequent modeling.

#### 2.9.3 Cell SA-*β*-gal staining

SA-β-gal staining was performed according to the instructions of the SA-*β*-gal staining kit (35). HepG2 cells were seeded in 6-well plates (Corning, USA) at 1 × 10^5^ cells per well and treated as follows: normal control, model (t-BHP), GL-BET (150 μg/mL), PB3 (150 μg/mL), PB4 (150 μg/mL), and positive control (10 μM resveratrol). After pretreatment, cells were exposed to t-BHP for 2 h, fixed, and stained with SA-β-gal solution (pH 6. 0) overnight at 37 °C in a CO₂-free incubator. Stained cells were imaged under a light microscope, and the percentage of blue-positive cells was calculated to assess senescence.

#### 2.9.4 Assessment of intracellular oxidative stress parameters

The intracellular ROS level was measured using the DCFH-DA fluorescent probe method (36). The intracellular concentrations of MDA, CAT, SOD, and GSH-Px were quantified using commercially available assay kits, strictly following the protocols.

#### 2.9.5 Western blot

Western blot analysis was performed as described previously (37).

#### 2.9.6 Statistical analysis

The response surface experimental data were processed by utilizing Design-Expert 13 software. The grayscale values of protein bands were analyzed with the use of ImageJ. Other data were processed by means of Excel 2024 software, and graphs were plotted using GraphPad Prism 10.1. 2 software. The results are presented as mean ± standard error (SEM). One-way analysis of variance (ANOVA) was employed to compare the differences between groups, and a *p*-value less than 0.05 was regarded as statistically significant.

## 3 Results

### 3.1 Comparison of extraction efficiencies of different deep eutectic solvents

Taking the reflux method with 70% ethanol as the control, the extraction effects of different solvents are shown in Table 1. Under fixed conditions (liquid-to-solid ratio 20 mL/g, water content 30%, 40 °C, 30 min), NADES 1 (GL-BET, 1:1) gave the highest proanthocyanidin yield (5.71%), 2. 72-fold higher than 70% ethanol (2.10%) (38). Extraction efficiency among HBDs followed: glycerol > DL-lactic acid > D-glucose > D-sorbitol, attributed to glycerol‘s three hydroxyl groups enabling a stable hydrogen-bond network with betaine (38). Increasing the betaine-to-HBD molar ratio from 1: 1 to 1: 2 significantly reduced yields, likely because excess betaine disrupts the hydrogen-bond network and lowers selectivity (27). Thus, GL-BET (1: 1) was selected as the optimal NADES system.

**Table 1:** Extraction effects of different extraction methods.

Solvent	HBD	HBA	Moisture content (%)	Molar ratio	Extraction yield (%)
NADES 1	Glycerol	Betaine	30%	1: 1	5. 71 ± 0.17
NADES 2	Glycerol			1: 2	1. 28 ± 0.03
NADES 3	D-Sorbitol			1: 1	1. 89 ± 0.06
NADES 4	D-Sorbitol			1: 2	0.27 ± 0.01
NADES 5	DL-Lactic acid			1: 1	4. 55 ± 0.18
NADES 6	DL-Lactic acid			1: 2	0.86 ± 0.04
NADES 7	D-Glucose			1: 1	3. 27 ± 0.09
NADES 8	D-Glucose			1: 2	0.68 ± 0.03
70% ethanol				/	2. 10 ± 0.10

### 3.2 Single-factor experiments on GL-BET extraction of LPPs

**Figure 1:**
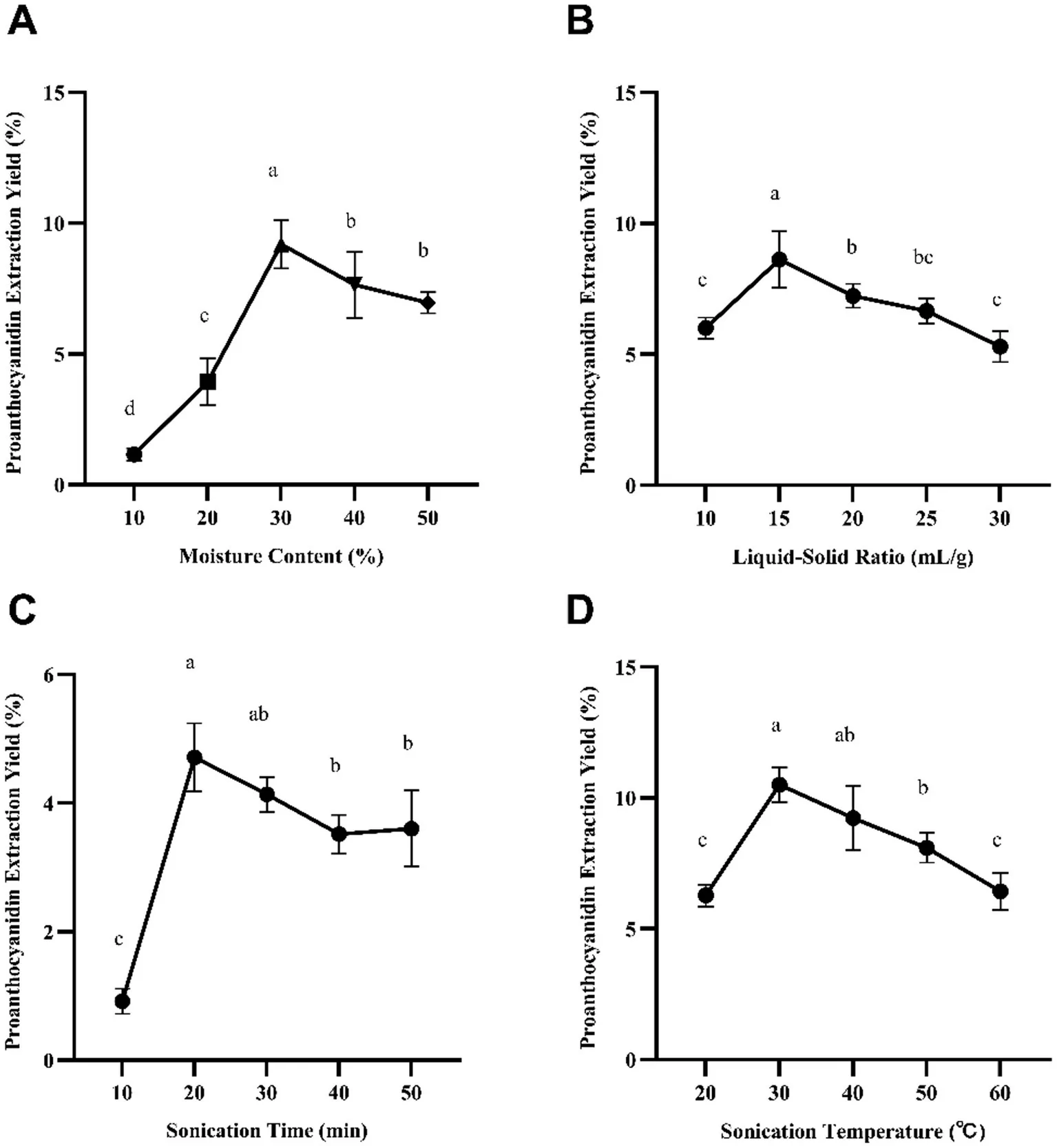
The influence of ultrasonic parameters on the extraction rate of LPPs: **(A)** water content; **(B)** liquid-to-solid ratio; **(C)** ultrasonic time; **(D)** ultrasonic temperature. The different letters in the data indicate the differences between the data sets, and the *p* < 0.05.

Figure 1A evaluated the effect of water content on the extraction yield. As the water content increased, the extraction yield initially rose, reaching a maximum of 9.19% at 30% water content, and then decreased at 40%. As shown in Figure 1B, the extraction yield increased with increasing liquid-to-solid ratio, peaking at 8.62% at 15 mL/g, after which further increases led to a decline. According to Figure 1C, within the sonication time range of 0–20 min, the extraction yield continuously increased, reaching a peak of approximately 4.81% at 20 min; beyond 20 min, the yield began to decrease. As illustrated in Figure 1D, when the extraction temperature was raised from 20 °C to 30 °C, the extraction yield gradually increased, reaching a maximum of 10.50% at 30 °C; further increasing the temperature to 40 °C resulted in a decrease in proanthocyanidin yield.

When the extraction temperature was increased from 20 °C to 30 °C, the yield of proanthocyanidins from lotus peel gradually increased, reaching its peak value (10.50%) at 30 °C. When the Sonication temperature was further raised to 40 °C, the proanthocyanidin yield decreased. The reason for this might be that a higher extraction temperature enhances the molecular movement speed and expedites solvent penetration, thus enabling proanthocyanidins from lotus peel to rapidly diffuse into the solvent. Nevertheless, an excessively high Sonication temperature causes the degradation of some proanthocyanidins, resulting in a reduction of the extraction yield (Figure 1D).

### 3.3 Response surface analysis and optimization

**Figure 2:**
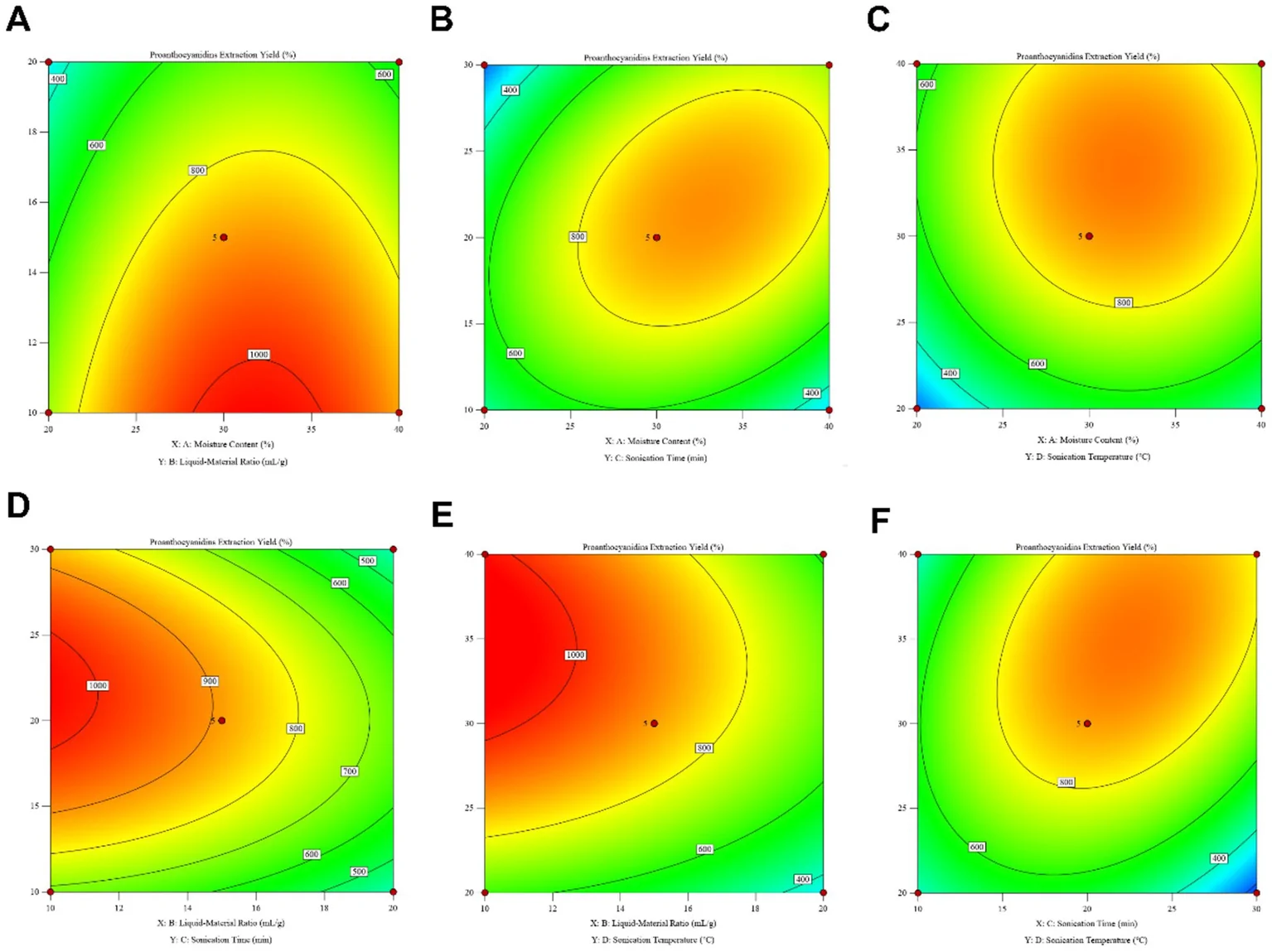
Two-dimensional contour map of the GL-BET extract, analyzing the influence of the interaction between independent variables on the extraction rate of LPPs: **(A)** AB; **(B)** AC; **(C)** AD; **(D)** BC; **(E)** BD; **(F)** CD. Green indicates the lowest response value, and the color changes as the value increases; deep red represents the highest response value obtained.

**Figure 3:**
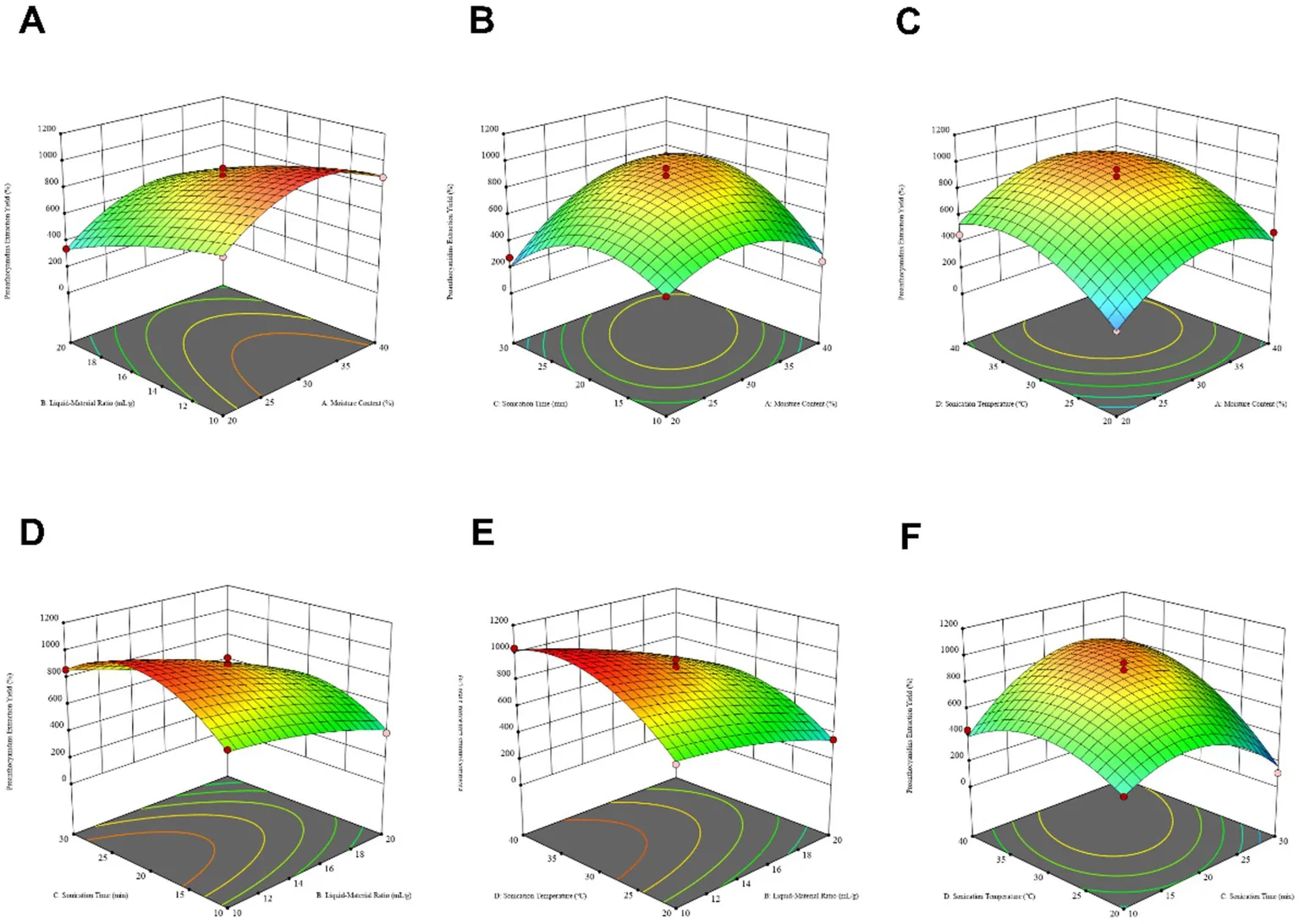
Three-dimensional response surface of the GL-BET extract, analyzing the influence of the interaction between independent variables on the extraction rate of LPPs: **(A)** AB; **(B)** AC; **(C)** AD; **(D)** BC; **(E)** BD; **(F)** CD. Green indicates the lowest response value, and the color changes as the value increases, deep red represents the highest response value obtained.

A quadratic polynomial regression model was constructed using water content (A), liquid-to-solid ratio (B), sonication time (C), and sonication temperature (D) as independent variables and proanthocyanidin yield (Y, % × 100) as the response variable based on the experimental data (Supplementary Table S2; Table 2). The regression equation was: Y = 888.75 + 94.59A-179.09B + 43.06C + 157.69D + 10.03AB + 160.31 AC-6.56 AD-45.75 BC-39.00BD + 161.25CD-221.21A^2^-45.72B^2^-253.80C^2^-198.11D^2^. As shown in Table 2, the model exhibited high significance (*p* < 0.01) and a non-significant lack-of-fit (*p* > 0.05), with R^2^ = 0.9805 and C. V.% = 8.49, indicating good fit and reliability. The order of influence on extraction yield was B (liquid-to-solid ratio) > D (temperature) > A (water content) > C (time). Elliptical contours for AC and CD interactions (*p* < 0.0001) confirmed synergistic effects (Figures 2, 3). As presented in Figures 2B,F, 3B,F, the response surfaces exhibited steep slopes and distinctly elliptical contours, consistent with the highly significant AC and CD terms. Within a certain range, appropriate water content and sonication time enhanced yield, while excessive time and temperature caused degradation. Optimal conditions predicted by response surface methodology were: water content of 34.97%, liquid-to-solid ratio of 10.07 mL/g, sonication time of 21.70 min, and sonication temperature of 35.47 °C, yielding a predicted extraction yield of 10.91%. Considering the limitations of actual experimental operations and instrumentation, the optimal parameters were adjusted to a water content of 35%, a liquid-to-solid ratio of 10 mL/g, a sonication time of 22 min, and a sonication temperature of 36 °C. Under these modified conditions, validation experiments were performed, and the measured proanthocyanidin yield was 10.97% (*n* = 3), which closely matched the theoretical predicted value, confirming the validity of the model.

**Table 2:** ANOVA results of regression equations.

Source	Sum of squares	Degree of freedom	Mean square	*F*-value	*p*-value	Significance
Model	1,773,000	14	126,700	50.29	<0.0001	**
A	107,400	1	107,400	42.64	<0.0001	**
B	384,900	1	384,900	152.83	<0.0001	**
C	22,252.55	1	22,252.55	8.84	0.0101	*
D	298,400	1	298,400	118.48	<0.0001	**
AB	402.5	1	402.5	0.16	0.6954	
AC	102,800	1	102,800	40.82	<0.0001	**
AD	172.27	1	172.27	0.07	0.7975	
BC	8,372.25	1	8,372.25	3.32	0.0897	
BD	6,084	1	6,084	2.42	0.1424	
CD	104,000	1	104,000	41.30	<0.0001	**
A^2^	317,400	1	317,400	126.04	<0.0001	**
B^2^	13,558.08	1	13,558.08	5.38	0.0359	*
C^2^	417,800	1	417,800	165.90	<0.0001	**
D^2^	254,600	1	254,600	101.08	<0.0001	**
Residual	35,258.82	14	2,518.49			
Lack of fit	29,633.82	10	2,963.38	2.11	0.2461	
Pure error	5,625	4	1,406.25			
Total	1,809,000	28				

Coefficient of determination R^2^ = 0.9805, C. V. % = 8.49.

### 3.4 Changes in the antioxidant activity of *in vitro* simulated digestion products

**Figure 4:**
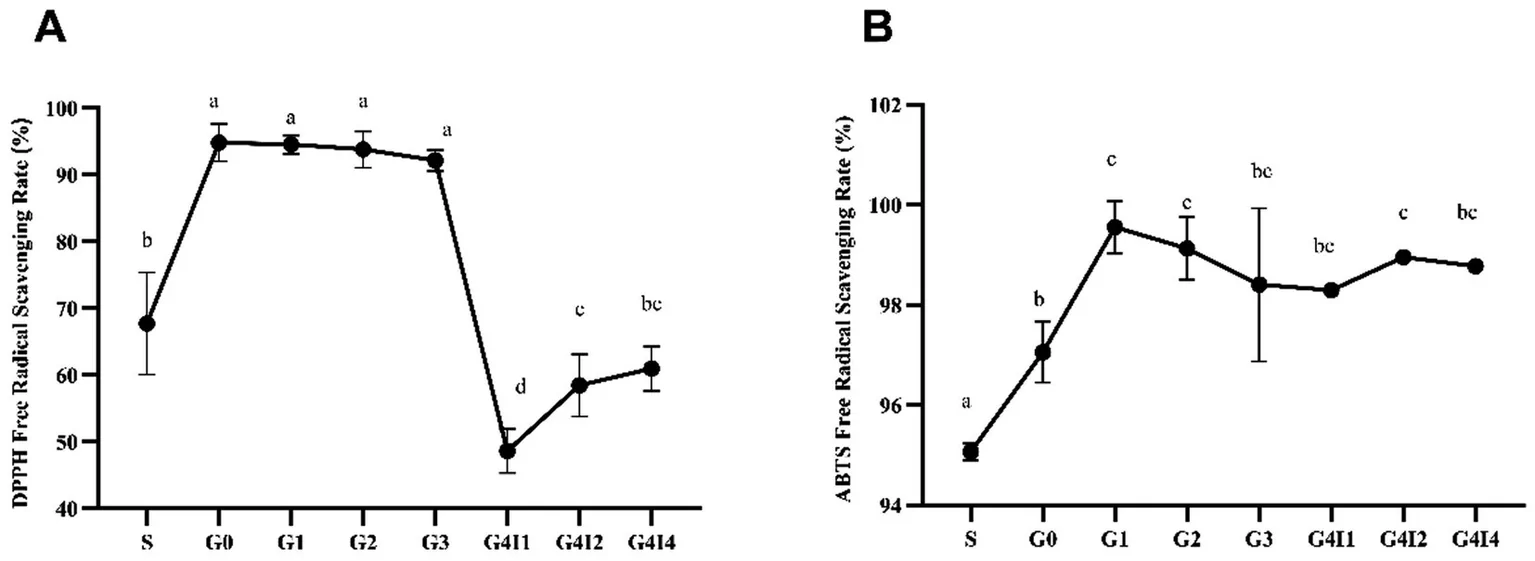
Simulated antioxidant capacity during each stage of digestion: **(A)** DPPH; **(B)** ABTS. Different letters in the data represent comparisons within the same column, indicating the differences between the data, with *p* < 0.05.

Figure 4A shows the DPPH radical scavenging activity of proanthocyanidins extracted from lotus peel using GL-BET at different digestion stages. During the oral digestion stage, the scavenging activity was at a moderate level. Upon entering the gastric digestion stage, the activity markedly increased and remained above 90% throughout the 4-h gastric digestion period. Compared with the end of gastric digestion, the scavenging activity decreased significantly during the intestinal stage. Notably, after 4 h of intestinal digestion, a partial recovery of DPPH scavenging activity was observed.

As shown in Figure 4B, the ABTS radical scavenging activity exhibited a similar stage-dependent trend to that of DPPH, albeit with relatively smoother fluctuations. Upon entering the intestinal digestion stage, the ABTS scavenging rate declined compared with that at the end of gastric digestion; however, the magnitude of this decline was smaller than that observed for DPPH scavenging activity.

### 3.5 Non-targeted metabolomics analysis

#### 3.5.1 Metabolite identification and composition analysis

**Figure 5:**
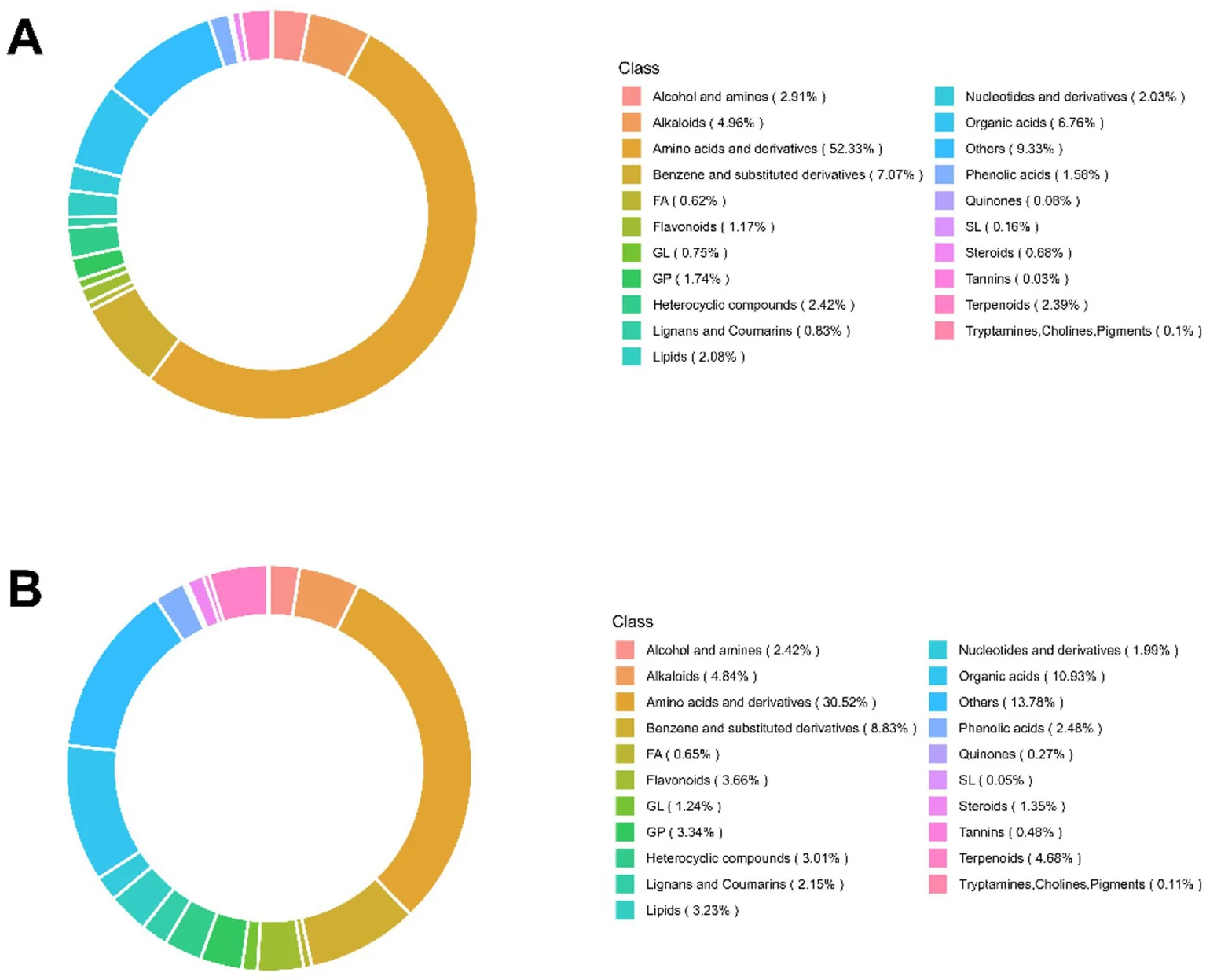
Circular diagram of metabolite category composition: **(A)** Positive ion mode; **(B)** Negative ion mode.

To gain a more comprehensive understanding of the metabolomic differences and metabolite variation patterns at various stages of *in vitro* simulated digestion of LPPs extracted with GL-BET, non-targeted metabolomics technology was utilized for metabolite analysis, as depicted in Figure 5. Ten samples from different stages were chosen, and three replicates were established for each stage in the metabolomic study. A total of 5,103 metabolites were detected, which included 2,302 amino acids and their derivatives, 393 benzene and its derivatives, 142 alcohols and amines, 96 phenolic acids, 48 glycerophospholipids, 48 glycerolipids, 96 nucleotides and their derivatives, 99 flavonoids, 8 quinones, 62 lignans and coumarins, 7 sphingolipids, 9 tannins, 5 tryptamines, cholines, and pigments, 240 alkaloids, 158 terpenoids, 433 organic acids, 131 heterocyclic compounds, 45 steroids, 33 fatty acyls, 123 lipids, and 555 other classes. Different colors correspond to different metabolite classes, and the area of each colored block reflects the relative proportion of that class.

#### 3.5.2 Sample quality control analysis

Quality control (QC) samples, prepared by pooling sample extracts and analyzed after every 10 test samples, exhibited consistent retention times and peak intensities in total ion current (TIC) chromatogram overlaps (Supplementary Figure S1), and Pearson correlation analysis among QC samples yielded correlation coefficients |r| ≥ 0.98 (Supplementary Figure S2), collectively demonstrating excellent instrument stability, data reliability, and repeatability.

#### 3.5.3 Metabolite screening and analysis

The fold-change threshold was set at FC ≥ 2 or FC ≤ 0.5. Comparisons were conducted between group S and groups G0, G1, G2, G3, G4I0, and G4I4, as well as between group G4I0 and groups G4I1, G4I2, and G4I4. The results indicated that the numbers of differential metabolites between group S and groups G0, G1, G2, G3, G4I0, and G4I4 were 3,763, 3,705, 3,727, 3,748, 3,942, and 3,958, respectively. Among these, the numbers of up-regulated metabolites were 2,054, 1,408, 1,288, 1,259, 1,527, and 1,488, respectively, while the numbers of down-regulated metabolites were 1,709, 2,297, 2,439, 2,489, 2,415, and 2,470, respectively. The numbers of differential metabolites between group G4I0 and groups G4I1, G4I2, and G4I4 were 1,768, 1,976, and 1,811, respectively. The numbers of up-regulated and down-regulated metabolites were as follows: 2,054, 1,408, 1,288, 1,259, 1,527, 1,488 up-regulated and 1,709, 2,297, 2,439, 2,489, 2,415, 2,470 down-regulated (Table 3).

**Table 3:** Statistics of the results of the metabolite.

Comparison group	Total number of differential metabolites	Number of upregulated differential metabolites	Number of down-regulated metabolites
G0 vs. S	3,763	2054	1709
S vs. G1	3,705	1,408	2,297
S vs. G2	3,727	1,288	2,439
S vs. G3	3,748	1,259	2,489
S vs. G4I0	3,942	1,527	2,415
S vs. G4I4	3,958	1,488	2,470
G4I0 vs. G4I1	1768	875	893
G4I0 vs. G4I2	1976	988	988
G4I0 vs. G4I4	1811	805	1,006

#### 3.5.4 Proanthocyanidin composition analysis

**Figure 6:**
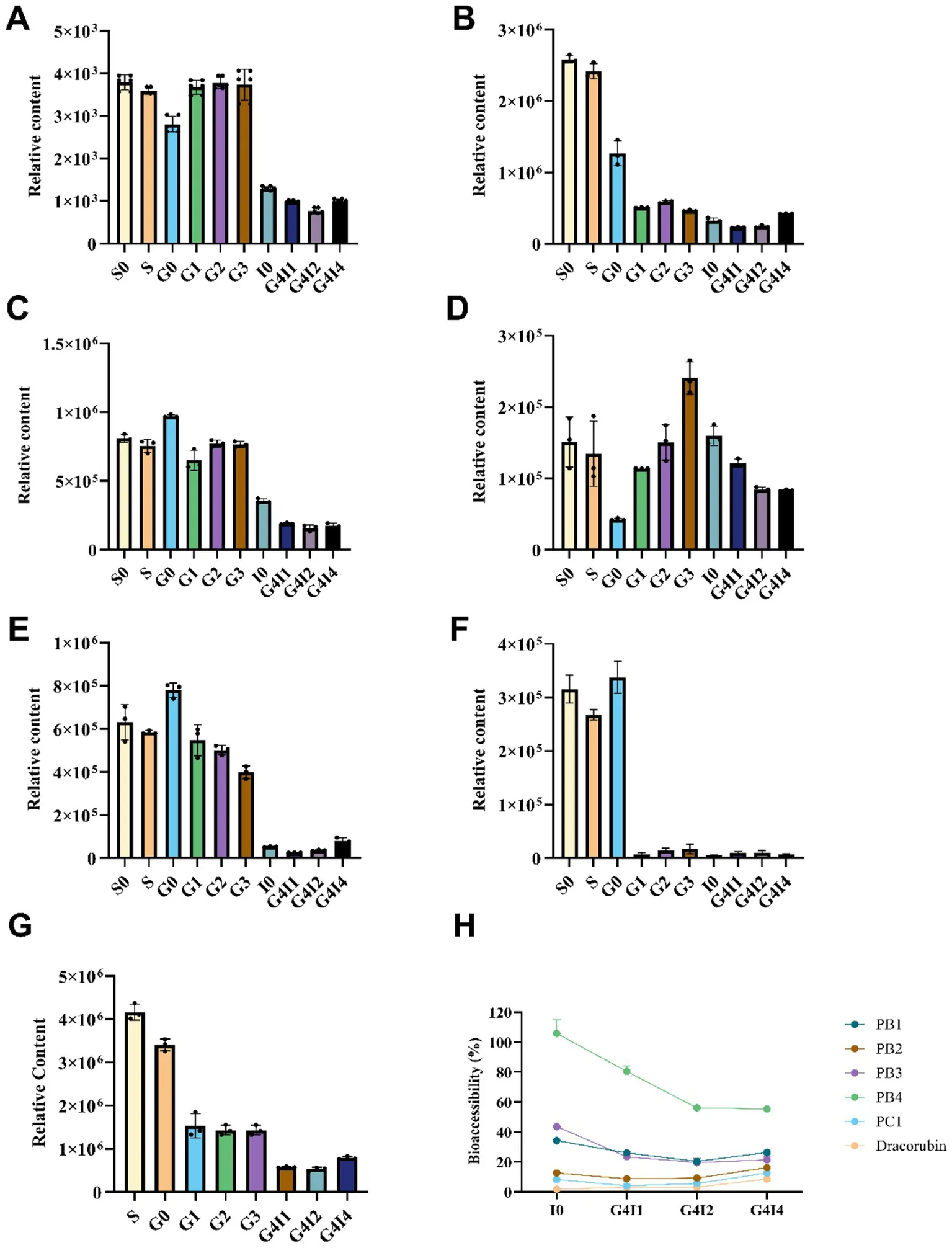
Untargeted profiling of proanthocyanidin contents and absorption rates during *in vitro* simulated digestion: **(A)** PB1; **(B)** PB2; **(C)** PB3; **(D)** PB4; **(E)** PC1; **(F)** Dracorubin; **(G)** Total proanthocyanidins; **(H)** Absorption effects of individual proanthocyanidins in the intestine.

After simulated digestion of LPPs extracted with GL-BET, a total of six proanthocyanidins were identified, as shown in Figure 6. During the digestion process, the total proanthocyanidin content generally decreased, with a pronounced decline during the gastric digestion phase, followed by a gradual increase during the intestinal digestion phase (Figure 6G). This phenomenon may be attributed to the combined effects of acid-induced chemical transformations and binding to pepsin during gastric digestion, leading to a significant reduction in total proanthocyanidin content, whereas the gradual increase during the intestinal phase reflects the synergistic contributions of pH-mediated dissociation, pancreatic enzyme hydrolysis, and bile salt solubilization effects (32, 39). As shown in Figures 6A–F, PB2 exhibited the highest content at the S0, S, G0, G4I1, G4I2, and G4I4 stages, while PB3 showed the highest content at the G1, G2, G3, and G4I0 stages, with PB1 consistently present at the lowest level throughout the entire digestion process. We hypothesize that dimers, owing to their moderate molecular size and favorable solubility stability, dominate at most digestion stages; the accumulation of trimers at specific stages, particularly in the early gastric phase, may reflect their role as intermediate products of high-molecular-weight polymer degradation (17, 40).

#### 3.5.5 Bioaccessibility during the *in vitro* simulated intestinal digestion stage

The bioaccessibility of proanthocyanidin monomers was assessed by tracking their relative content changes during intestinal digestion (Figure 6H). All six monomers declined to varying extents; PB4 retained the highest relative content (approximately 50%) after 4 h, followed by PB2 and PB3, whereas PB1, PC1, and dracorubin were more extensively degraded. The strong intestinal stability of PB4 is attributed to its B-type dimer structure and specific B-ring hydroxylation pattern, which limit enzymatic recognition and hydrolysis under weakly alkaline conditions (41). PB3, owing to its lower polymerization degree, is more prone to oxidation or bile-salt precipitation, leading to a lower retention than PB4 (42). PB1 declined rapidly, possibly due to selective degradation or absorption, while PC1 and dracorubin (high molecular weight) were readily degraded into phenolic acids or monomers (17, 43).

Based on their favorable stability and bioaccessibility, PB3 and PB4 were selected for further mechanistic studies.

### 3.6 Cytotoxicity and cytoprotection of GL-BET, PB3, and PB4 in HepG2 cell**s**

**Figure 7:**
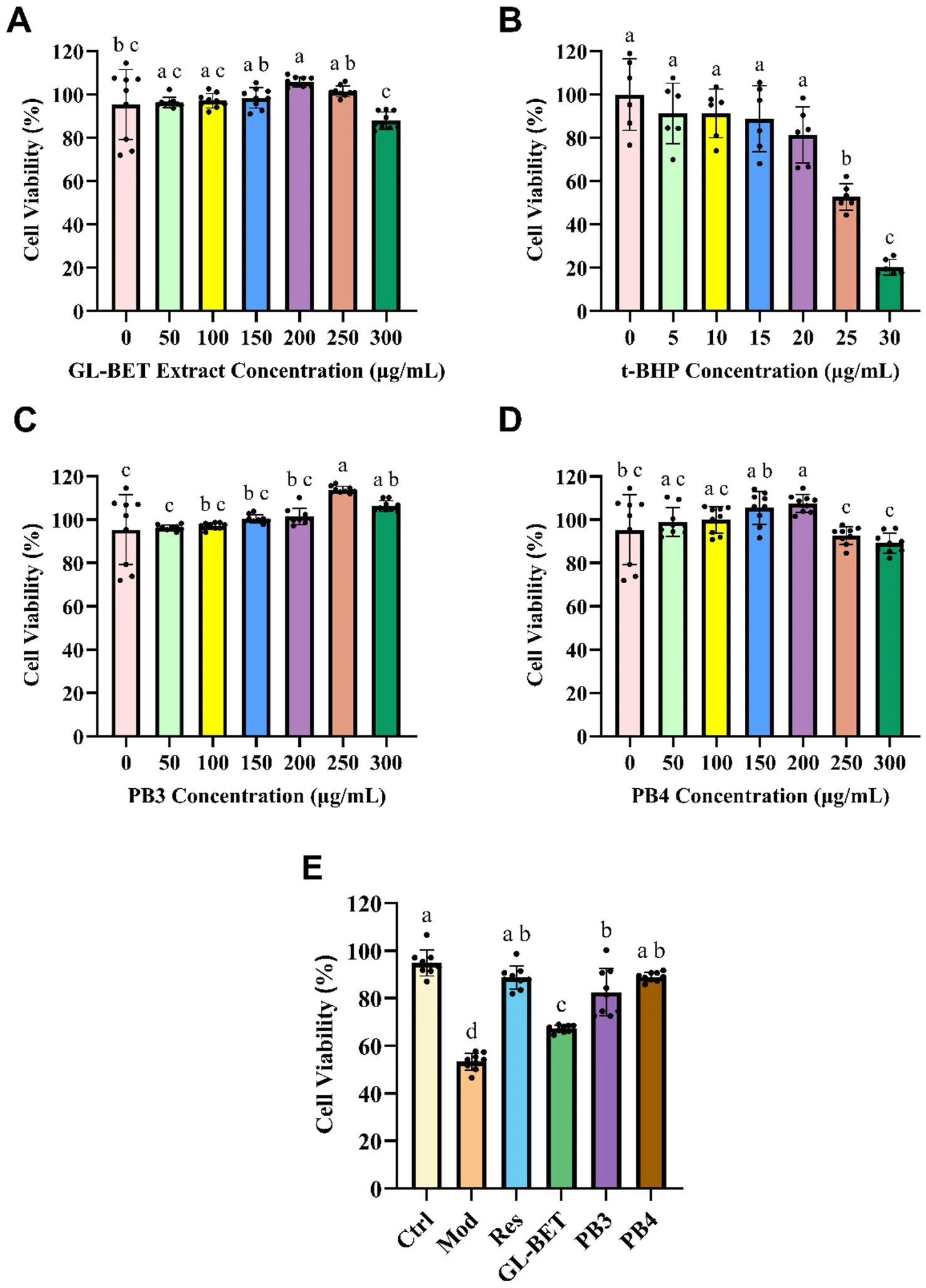
Cytotoxic effects of proanthocyanidins and t-BHP on HepG2 cells **(A–D)**, and protective effects of proanthocyanidins against t-BHP-induced oxidative injury in HepG2 cells **(E)**. Different letters in the data represent comparisons within the same column, indicating the differences between the data, with *p* < 0.05.

To determine the safe working concentrations of GL-BET, PB3, and PB4, HepG2 cells were treated with 0–300 μg/mL of each compound for 24 h, and cell viability was measured using the CCK-8 assay (Figures 7A–D). Within 0–150 μg/mL, no significant inhibitory effect on cell viability was observed (*p* > 0.05), with survival rates above 95%; at some concentrations, a mild pro-proliferative effect was noted (44). At ≥200 μg/mL, viability in the GL-BET and PB4 groups declined but remained around 85%. To avoid nonspecific cytotoxicity, 150 μg/mL was selected as the safe working concentration for subsequent experiments (45), and 10 μM resveratrol served as the positive control (46). To establish a stable oxidative stress-induced senescence model, HepG2 cells were exposed to gradient concentrations of t-BHP (0–30 μg/mL) for 2 h. As shown in Figure 7D, cell viability decreased in a concentration-dependent manner, dropping to approximately 52.73% at 25 μg/mL; therefore, 25 μg/mL t-BHP was chosen for model induction (47–49). Under these conditions, the model group (t-BHP-treated HepG2 cells) exhibited significantly lower viability than the blank control group (*p* < 0.05), confirming successful establishment of the senescence model (Figure 7E). Compared with the model group, treatment with GL-BET, PB3, PB4, or resveratrol significantly increased cell viability (*p* < 0.05), indicating that these compounds effectively counteract t-BHP-induced oxidative stress and protect cell viability (50, 51). Among the three tested compounds in cellular assays (GL-BET, PB3, and PB4), PB4 consistently exhibited the strongest cytoprotective effect, comparable to those of the positive control resveratrol (52).

### 3.7 Effect on SA-*β*-gal activity and intracellular ROS levels

**Figure 8:**
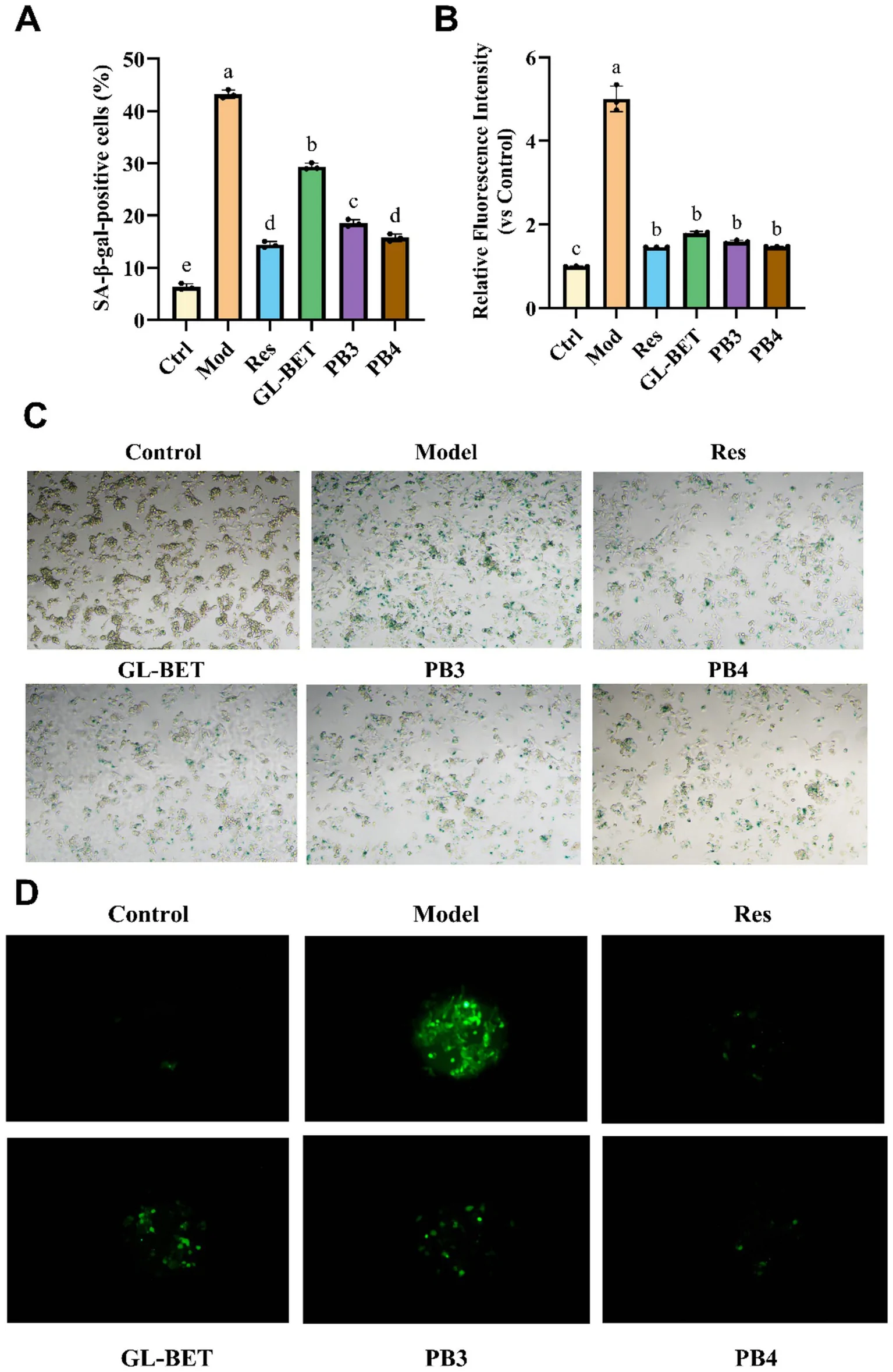
SA-*β*-gal activity and intracellular ROS levels in HepG2 cells. **(A)** Proportion of SA-β-gal-positive cells; **(B)** Relative ROS content; **(C)** Representative images of SA-β-gal staining (×10); **(D)** Fluorescence images of ROS (×20). Different letters within the same column indicate significant differences (*p* < 0.05).

SA-β-galactosidase staining was performed on HepG2 cells. Representative images and quantitative positive rates are shown in Figures 8A,C. In the normal control group, very few SA-β-gal-positive cells were observed (<10%), and the cells maintained a regular morphology. Following t-BHP treatment, the model group exhibited a marked increase in the positive rate to approximately 43.23% (*p <* 0.001), accompanied by enlarged and flattened cell morphology, indicating a typical senescent phenotype. Compared with the model group, pretreatment with GL-BET, PB3, PB4, or resveratrol significantly reduced the positive rates to approximately 29.37, 18.52, 15.76, and 14.42%, respectively (*p <* 0.001). Notably, the positive rate in the PB4 group was not statistically different from that in the resveratrol group (*p* > 0.05) but was significantly lower than that in the PB3 group (*p* < 0.05). Simultaneously, intracellular ROS levels were detected using the DCFH-DA fluorescent probe. Representative fluorescence images and the statistical results of relative fluorescence intensity are presented in Figures 8B,D. Compared with the control group, t-BHP-stimulated HepG2 cells showed significantly brighter green fluorescence, indicating cellular oxidative damage. In contrast, the fluorescence intensity was markedly attenuated in groups pretreated with GL-BET, PB3, PB4, or resveratrol. Figure 8B revealed that the model group had significantly higher ROS levels than the control group (*p* < 0.05), confirming successful establishment of the oxidative injury model. Treatment with GL-BET, PB3, or PB4 all significantly reduced ROS levels (*p* < 0.05), consistent with the trend observed in the resveratrol group. The efficacy in reducing ROS levels followed the order from weakest to strongest: GL-BET, PB3, and PB4, with PB4 achieving a level close to that of resveratrol. Collectively, these results demonstrate that t-BHP successfully establishes a cellular senescence model, and that GL-BET, PB3, and PB4 effectively delay t-BHP-induced senescence and ameliorate oxidative damage in HepG2 cells.

### 3.8 Effects of GL-BET, PB3, and PB4 on oxidative stress markers in t-BHP-induced HepG2 cells

**Figure 9:**
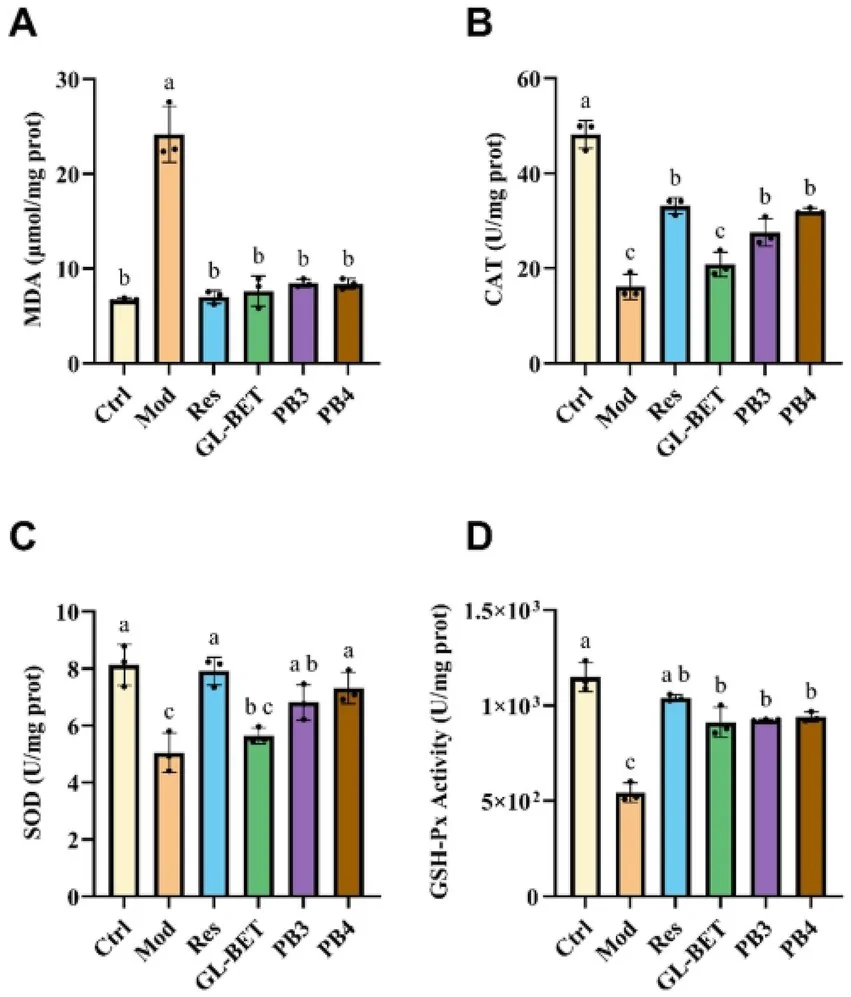
Analysis of oxidative stress indicators in HepG2 cells. **(A)** MDA; **(B)** CAT; **(C)** SOD; **(D)** GSH-Px. Different letters in the data represent comparisons within the same column, indicating the differences between the data, with *p* < 0.05.

To investigate the intervention effects of GL-BET, PB3, and PB4 on cellular oxidative damage, the content of the lipid peroxidation product malondialdehyde (MDA) and the activities of key antioxidant enzymes (SOD, CAT, and GSH-Px) were measured. The results are shown in Figure 9A. The MDA content in the model group was significantly higher than that in the control group (*p* < 0.001), indicating that t-BHP treatment induced severe lipid peroxidation injury. Compared with the model group, pretreatment with GL-BET, PB3, PB4, or resveratrol significantly reduced the cellular MDA content (*p* < 0.05 or *p* < 0.01).

The activities of SOD, CAT, and GSH-Px in the model group were significantly lower than those in the control group (*p* < 0.001), indicating that t-BHP suppressed the endogenous antioxidant enzyme system (Figures 9B–D). Compared with the model group, GL-BET treatment partially restored the activities of these antioxidant enzymes, and PB4 treatment exhibited a more pronounced effect, comparable to that of resveratrol. Moreover, the antioxidant enzyme activities in the PB4 group were significantly higher than those in the GL-BET group (*p* < 0.05). These results demonstrate that GL-BET, PB3, and PB4 alleviate t-BHP-induced oxidative damage by restoring antioxidant enzyme activities and inhibiting lipid peroxidation.

### 3.9 Nrf2/Keap1/ARE signaling in cellular anti-senescence

**Figure 10:**
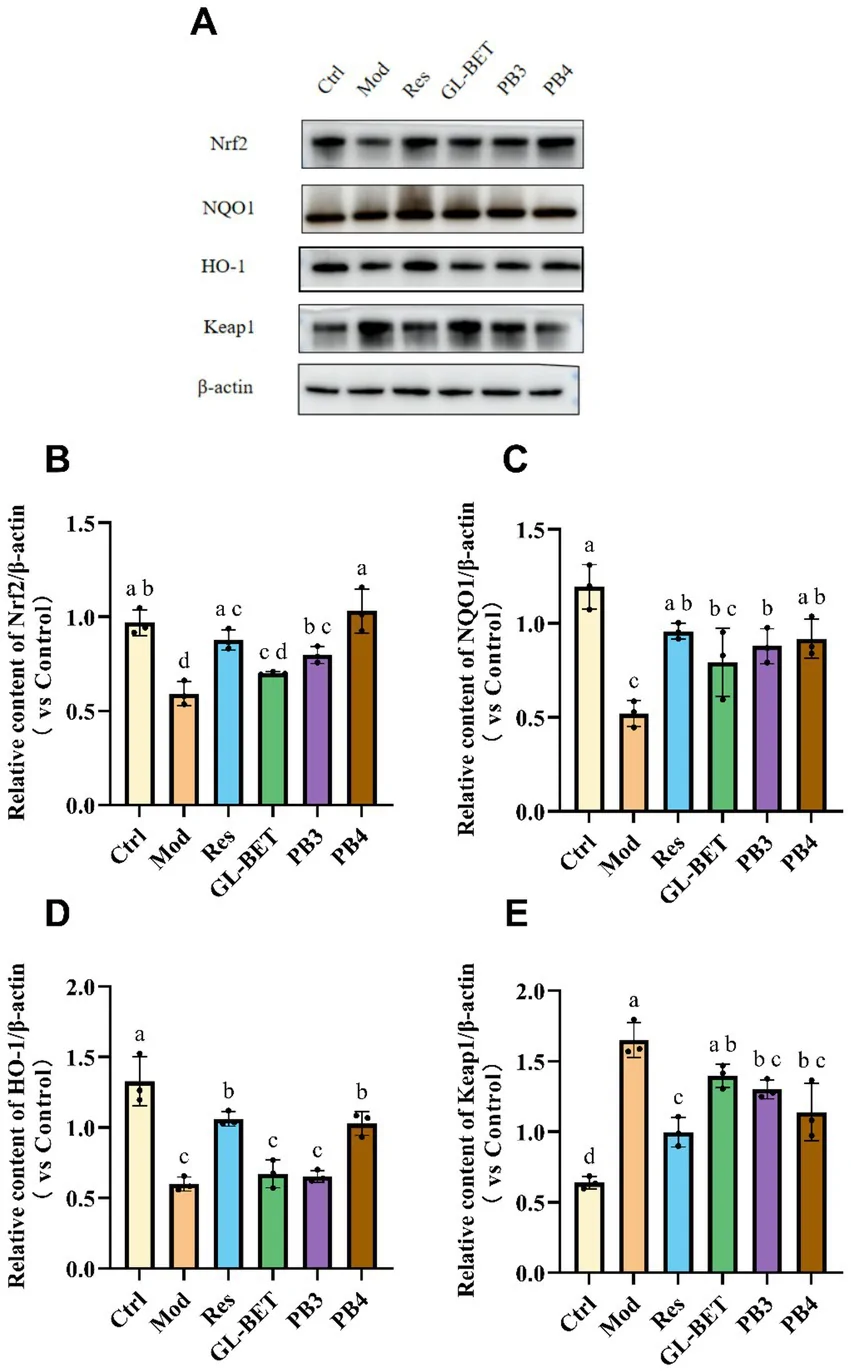
Effects of three substances at the same concentration on protein expression in HepG2 cells during oxidative stress. **(A)** The stripes of protein expression; **(B)** Nrf2; **(C)** NQO1; **(D)** HO-1; **(E)** Keap1. Different letters in the data represent comparisons within the same column, indicating the differences between the data, with *p* < 0.05.

To elucidate the molecular mechanisms underlying the antioxidant and anti-aging effects of GL-BET, PB3, and PB4, the protein expression levels of the anti-aging-related proteins Nrf2, Keap1, HO-1, and NQO1 were examined by Western blotting. The results are presented in Figure 10. In HepG2 cells, the protein expression levels of Nrf2, NQO1, and HO-1 were all significantly decreased (*p* < 0.001), whereas Keap1 expression was markedly increased (*p* < 0.01), indicating that t-BHP disrupted the Nrf2/Keap1/ARE antioxidant defense axis. Pretreatment with GL-BET significantly upregulated the expression of Nrf2, NQO1, and HO-1 (*p* < 0.05). Among the tested compounds, PB4 exhibited the most potent effect, comparable to that of the positive control resveratrol. Collectively, these results demonstrate that GL-BET, PB3, and PB4 effectively counteract t-BHP-induced oxidative stress injury by inhibiting Keap1 expression, promoting Nrf2 nuclear translocation, and consequently activating the downstream expression of HO-1 and NQO1. Among the three tested compounds in cellular assays (GL-BET, PB3, and PB4), PB4 consistently exhibited the strongest regulatory capacity on this signaling pathway.

## 4 Discussion

In this study, an ultrasound-assisted natural deep eutectic solvent (NADES) extraction method was developed for the efficient recovery of proanthocyanidins from lotus peel, a low-value by-product of lotus seed processing. Among the eight NADES systems tested, betaine-glycerol (GL-BET) at a 1: 1 molar ratio gave the highest extraction yield (5. 71%), which was 2. 72-fold higher than that obtained with conventional 70% ethanol reflux extraction (2. 10%). The superiority of GL-BET can be attributed to the high hydroxyl density and low steric hindrance of glycerol, which together with betaine forms a stable hydrogen-bond network that is highly compatible with the polyphenolic structure of proanthocyanidins (38, 53). Recent molecular dynamics simulations have elucidated that the enhanced extraction efficiency of NADES arises from higher solvent-accessible surface area values, a greater number of hydrogen bonds, extended hydrogen bonding lifetimes, and lower intermolecular interaction energies between NADES and proanthocyanidins (54). These findings are consistent with a previous report showing that ultrasound-assisted DES extraction achieved a yield of 78.58 mg/g from cottonseed hulls, with molecular dynamics simulations revealing stronger van der Waals forces and hydrogen-bonding interactions between DES and catechin compared to conventional solvents (16). When the molar ratio of betaine to HBD was increased to 1: 2, extraction yields dropped sharply, likely because excess betaine occupies hydrogen-bond donor sites and disrupts the solvent’s selective solubility for proanthocyanidins (27). These findings are consistent with previous reports showing that NADES can outperform conventional organic solvents in extracting phenolic compounds from plant matrices (55). Moreover, the molar ratio of hydrogen bond acceptors to donors in NADES has a crucial impact on the extraction efficiency (56).

Response surface methodology (RSM) was then employed to optimize the extraction parameters. The model showed excellent fit (R^2^ = 0.9805) and identified water content (A), liquid-to-solid ratio (B), sonication time (C) and sonication temperature (D) as significant factors, with the order of influence B > D > A > C. The interaction terms AC (water content × sonication time) and CD (sonication time × temperature) were highly significant (*p* < 0.0001), indicating synergistic effects. The optimal conditions (water content 35%, liquid-to-solid ratio 10 mL/g, sonication time 22 min, temperature 36 °C) gave an experimental yield of 10.97%, which agreed well with the predicted value (10.91%). This yield represents a 5. 23-fold improvement over ethanol reflux extraction, demonstrating that NADES-based ultrasound extraction is both efficient and sustainable. Similar improvements have been reported for other plant matrices, such as cottonseed hulls and Korean pine bark (16, 57).

After establishing the optimal extraction procedure, the digestive fate and bioaccessibility of the GL-BET extract were investigated using a standardized INFOGEST static *in vitro* digestion model combined with untargeted metabolomics. The DPPH and ABTS radical scavenging activities of the extract exhibited stage-dependent changes. During gastric digestion, DPPH scavenging increased markedly (by 17.01–41.48% relative to the oral phase) and remained above 90% throughout the 4 h gastric period, whereas it declined by 26. 03–49.75% upon entering the intestinal phase. The ABTS scavenging showed a more gradual change, with a smaller decrease in the intestinal phase. These differences can be explained by the distinct mechanisms of the two assays: DPPH scavenging relies primarily on hydrogen atom transfer (HAT), which is highly sensitive to conformational changes of phenolic compounds, whereas ABTS scavenging can proceed via both HAT and single electron transfer (SET), conferring greater adaptability (32). The increase in antioxidant activity during gastric digestion may be due to acid-induced exposure of hydroxyl groups and partial depolymerisation of high-molecular-weight proanthocyanidins into smaller oligomers with enhanced hydrogen-donating capacity (39, 58). Conversely, the decline in the intestinal phase is likely caused by the weakly alkaline environment (which deprotonates phenolic hydroxyls), complexation with bile salts, and enzymatic degradation (59, 60). Notably, a partial recovery of DPPH scavenging was observed after 4 h of intestinal digestion, suggesting that some degradation products (e. g., phenolic acids) retain radical-scavenging activity (43).

Untargeted metabolomics identified six proanthocyanidin monomers: PB1, PB2, PB3, PB4, PC1 and dracorubin. Total proanthocyanidin content decreased during gastric digestion but increased again in the intestinal phase, reflecting pH-mediated dissociation and bile salt solubilization (17, 59). PB2 was the predominant monomer in the oral and late-intestinal phases, whereas PB3 peaked during gastric digestion, supporting the view that dimers and trimers may act as intermediate products of polymer degradation (40). Among all monomers, PB4 showed the highest retention (55. 32%) after 4 h of intestinal digestion, followed by PB2 and PB3, while PB1, PC1 and dracorubin were more rapidly degraded. The superior intestinal stability of PB4 can be linked to its B-type dimer structure with a specific B-ring hydroxylation pattern that limits enzymatic recognition and hydrolysis (41, 42). Based on their favorable stability and bioaccessibility, PB3 and PB4 were selected for subsequent cellular mechanistic studies.

At the cellular level, the GL-BET extract, PB3 and PB4 were non-cytotoxic to HepG2 cells at concentrations up to 150 μg/mL. A t-BHP concentration of 25 μg/mL (2 h exposure) reduced cell viability to approximately 52.73%, establishing a reliable oxidative stress-induced premature senescence model (48, 49). Pretreatment with 150 μg/mL of each compound significantly restored viability, with PB4 showing the strongest rescue effect (88.98% viability). This cytoprotection was accompanied by a marked reduction in SA-*β*-gal-positive cells (from 43.23 to 15.76%), indicating that the compounds effectively suppress cellular senescence. Moreover, GL-BET, PB3 and PB4 dose-dependently reduced intracellular ROS levels and MDA content, while enhancing the activities of the key antioxidant enzymes SOD, CAT and GSH-Px. These results are consistent with the well-established antioxidant properties of proanthocyanidins (52, 61).

To elucidate the molecular mechanism underlying the antioxidant and anti-aging effects, western blotting was performed to assess the Nrf2/Keap1/ARE pathway. In t-BHP-treated cells, Keap1 expression was upregulated, while Nrf2, HO-1 and NQO1 were downregulated, indicating that oxidative stress disrupts this critical cytoprotective axis. Pretreatment with GL-BET, PB3 or PB4 reversed these changes: Keap1 was suppressed, and the expression of Nrf2, HO-1 and NQO1 was restored. Among the three, PB4 showed the strongest regulatory potency, similar to that of resveratrol. The Nrf2/Keap1/ARE pathway is a master regulator of cellular antioxidant defense; its activation leads to the transcription of phase II detoxifying and antioxidant enzymes such as HO-1 and NQO1 (62). The ability of PB4 to activate this pathway explains its superior cytoprotective, antioxidant and anti-inflammatory effects. These results are in agreement with recent reports that certain B-type procyanidin dimers can act as Nrf2 activators in various cell models (51, 63). For instance, procyanidin B2 has been shown to activate the Keap1/Nrf2/HO-1 signaling pathway and protect cells against oxidative damage, and to alleviate liver injury through activation of Nrf-2/Keap1 and AMPK/GSK3β signaling pathways. Procyanidin A1 and its digestive products have also been reported to alleviate cell damage through regulating the Keap1/Nrf2 pathway. Furthermore, a recent study demonstrated that proanthocyanidins from lotus seed skin mitigate glycolipid metabolism disorder through the p38/Nrf2/NF-κB signaling pathway. The Nrf2/Keap1 pathway can be activated by natural products through both non-covalent and covalent modulation mechanisms, with polyphenols representing a major class of Keap1 modifiers (56). Huang et al. (64) further demonstrated that proanthocyanidins regulate ovarian oxidative stress through the SESTRIN2-NRF2 pathway, promoting NRF2 and Keap1 separation and subsequent nuclear translocation, where NRF2 promotes the expression of a variety of cytoprotective genes (64).

Interestingly, at concentrations between 50 and 100 μg/mL, we observed a mild but reproducible increase in cell viability (approximately 5–10% above the control), suggesting a possible hormetic effect of these proanthocyanidins. Such biphasic dose-responses are common for plant-derived polyphenols, which at low doses can activate pro-survival pathways or improve mitochondrial bioenergetics, thereby promoting cell proliferation. Similar observations have been reported for NADES-based broccoli extracts, which exhibited mild stimulation of HaCaT cell growth (65). The proliferation-promoting effect disappeared at higher concentrations (≥200 μg/mL), indicating that the hormetic window is narrow and concentration-dependent. This phenomenon supports the safety of our chosen working concentration (150 μg/mL) and underscores the importance of dose optimization in the application of natural antioxidants.

Taken together, our results demonstrate that GL-BET is an efficient and green solvent for extracting proanthocyanidins from lotus peel. The extract and its characteristic monomers PB3 and PB4, especially PB4, exert potent anti-aging effects by activating the Nrf2/Keap1/ARE pathway, thereby enhancing endogenous antioxidant enzyme activities, reducing ROS and lipid peroxidation, and suppressing pro-inflammatory cytokines. The high intestinal bioaccessibility and stability of PB4 further support its potential as a functional food ingredient for delaying aging and preventing age-related diseases. Future studies should focus on *in vivo* validation using animal models of aging, as well as pharmacokinetic investigations to assess the systemic bioavailability and tissue distribution of PB4 following oral administration.

## 5 Conclusion

In this study, an ultrasound-assisted natural deep eutectic solvent (NADES) method using betaine-glycerol (GL-BET, 1:1) was developed for the green and efficient extraction of proanthocyanidins from lotus peel. Under the optimized conditions determined by response surface methodology (35% water content, liquid-to-solid ratio of 10 mL/g, sonication time of 22 min at 36 °C), a proanthocyanidin yield of 10.97% was achieved, which was 5.23-fold higher than that obtained with conventional 70% ethanol reflux. This result underscores the potential of GL-BET as a sustainable solvent for valorizing lotus peel by-products.

Using an INFOGEST-compliant *in vitro* digestion model combined with untargeted metabolomics, six proanthocyanidin monomers (PB1, PB2, PB3, PB4, PC1, and dracorubin) were identified. Notably, PB4 exhibited the highest intestinal stability, retaining 55.32% of its initial concentration after 4 h of intestinal digestion, suggesting its potential for sustained bioactivity in the gastrointestinal tract.

Furthermore, the anti-senescence potential of GL-BET, PB3, and PB4 was systematically evaluated in a t-BHP-induced HepG2 cell aging model. All three compounds were non-cytotoxic at concentrations ≤150 μg/mL, significantly improved cell viability, reduced the proportion of SA-*β*-gal-positive cells, decreased intracellular ROS and MDA levels, and restored the activities of SOD, CAT, and GSH-Px. Mechanistic investigations revealed that these proanthocyanidins counteract oxidative stress-induced senescence through modulation of the Nrf2/Keap1/ARE signaling pathway, specifically by downregulating Keap1 and upregulating Nrf2, HO-1, and NQO1. Among the three tested compounds in cellular assays (GL-BET, PB3, and PB4), PB4 consistently exhibited the strongest anti-senescence effects, comparable to those of the positive control resveratrol.

In summary, our findings establish GL-BET as an efficient and environmentally friendly solvent for extracting LPPs and identify PB4 as a promising natural anti-aging agent acting via the Nrf2/Keap1/ARE pathway. This work provides a novel strategy for the high-value utilization of lotus peel agricultural by-products and lays a foundation for the development of proanthocyanidin-enriched functional foods aimed at combating aging and oxidative stress-related diseases.

## Data Availability

The original contributions presented in the study are included in the article/Supplementary material, further inquiries can be directed to the corresponding authors.
