# Supplementary material for: Deep eutectic solvent extraction of lotus peel proanthocyanidins and its anti-senescence mechanism in t-BHP induced HepG2 cells
Source: Front Nutr. 2026 Aug 5;13:1884044. doi: 10.3389/fnut.2026.1884044 (PMC13495210; doi:10.3389/fnut.2026.1884044)
Supplement: Supplementary file 1 [file Supplementary_file_1.DOCX]

Supplementary Material

**Table S1 Design table of factor levels for response surface experiment optimization**

| Level | A Moisture content  (%) | B Liquid-to-solid ratio  (mL/g) | C Sonication time  (min) | D Sonication temperature  (℃) |
| --- | --- | --- | --- | --- |
| -1 | 20 | 10 | 10 | 20 |
| 0 | 30 | 15 | 20 | 30 |
| 1 | 40 | 20 | 30 | 40 |

**Table S2 Levels design table of optimization factors for response surface test**

| Number | Moisture content  (A) | Liquid-to-solid ratio  (B) | Sonication Duration  (C) | Sonication temperature  (D) | Extraction yield(%) |
| --- | --- | --- | --- | --- | --- |
| 1 | 20 | 10 | 20 | 30 | 7. 09 |
| 2 | 40 | 10 | 20 | 30 | 8. 81 |
| 3 | 20 | 20 | 20 | 30 | 3. 42 |
| 4 | 40 | 20 | 20 | 30 | 5. 55 |
| 5 | 30 | 15 | 10 | 20 | 4. 01 |
| 6 | 30 | 15 | 30 | 20 | 1. 09 |
| 7 | 30 | 15 | 10 | 40 | 4. 43 |
| 8 | 30 | 15 | 30 | 40 | 7. 95 |
| 9 | 20 | 15 | 20 | 20 | 2. 10 |
| 10 | 40 | 15 | 20 | 20 | 4. 76 |
| 11 | 20 | 15 | 20 | 40 | 4. 58 |
| 12 | 40 | 15 | 20 | 40 | 6. 98 |
| 13 | 30 | 10 | 10 | 30 | 6. 94 |
| 14 | 30 | 20 | 10 | 30 | 3. 90 |
| 15 | 30 | 10 | 30 | 30 | 8. 62 |
| 16 | 30 | 20 | 30 | 30 | 3. 75 |
| 17 | 20 | 15 | 10 | 30 | 4. 46 |
| 18 | 40 | 15 | 10 | 30 | 2. 48 |
| 19 | 20 | 15 | 30 | 30 | 2. 78 |
| 20 | 40 | 15 | 30 | 30 | 7. 20 |
| 21 | 30 | 10 | 20 | 20 | 6. 08 |
| 22 | 30 | 20 | 20 | 20 | 3. 53 |
| 23 | 30 | 10 | 20 | 40 | 10.34 |
| 24 | 30 | 20 | 20 | 40 | 6. 23 |
| 25 | 30 | 15 | 20 | 30 | 8. 51 |
| 26 | 30 | 15 | 20 | 30 | 9. 49 |
| 27 | 30 | 15 | 20 | 30 | 8. 66 |
| 28 | 30 | 15 | 20 | 30 | 8. 96 |
| 29 | 30 | 15 | 20 | 30 | 8. 81 |

**Table S3 1.25×Electrolyte solution reserve formula**

| Reagent Name | Concentration(M) | SGF Concentration(mM) | SIF Concentration(mM) |
| --- | --- | --- | --- |
| KCl | 0.50 | 6.90 | 6.8 |
| KH_2_PO_4_ | 0.50 | 0.90 | 0.8 |
| NaHCO_3_ | 1.00 | 25.00 | 85.00 |
| NaCl | 2.00 | 47.20 | 38.40 |
| MgCl_2_(H_2_O)_6_ | 0.15 | 0.12 | 0.33 |
| (NH4)_2_CO_3_ | 0.50 | 0.50 | - |
| HCl | 6.00 | 15.60 | 8.40 |
| CaCl_2_(H_2_O)_2_ | 0.30 | 0.15 | 0.60 |

**Table S4 Sample identification information**

| Number | Phase |
| --- | --- |
| S_0_ | Pre-digestion |
| S | Oral digestion (5 min) |
| G_0_ | Initial entry into the stomach |
| G_1_ | Gastric digestion (1 h) |
| G_2_ | Gastric digestion (2 h) |
| G_3_ | Gastric digestion (4 h) |
| G_4_I_0_ | Initial entry into the intestine after 4 h of gastric digestion |
| G_4_I_1_ | 4 h gastric digestion + 1 h intestinal digestion |
| G_4_I_2_ | 4 h gastric digestion + 2 h intestinal digestion |
| G_4_I_4_ | 4 h gastric digestion + 4 h intestinal digestion |

**Table S5 Gradient conditions of mobile phase for T3 chromatography column**

| Time (min) | A (%) | B (%) |
| --- | --- | --- |
| 0.0 | 95 | 5 |
| 5.0 | 35 | 65 |
| 6.0 | 1 | 99 |
| 7.5 | 1 | 99 |
| 7.6 | 95 | 5 |
| 10.0 | 95 | 5 |

**Table S6 AB TripleTOF 6600 mass spectrometry conditions**

| Instrument | ESI+ | ESI- |
| --- | --- | --- |
| Duration (min) | 10 | 10 |
| IonSpray Voltage (V) | 5000 | -4000 |
| Temperature (°C) | 550 | 550 |
| Ion Source Gas1 (psi) | 50 | 50 |
| Ion Source Gas2 (psi) | 60 | 60 |
| Curtain Gas (psi) | 35 | 35 |
| Declustering Potential (V) | 80 | -80 |
| MS1 Collision Energy (V) | 10 | -10 |
| MS2 Collision Energy (V) | 30 | -30 |
| Collision Energy Spread (V) | 15 | 15 |
| MS1 TOF Masses (Da) | 50~1250 | 50~1250 |
| MS2 TOF Masses (Da) | 25~1250 | 25~1250 |

**Figure S1 TIC overlay of QC sample mass spectrometry detection: (A) Positive ion mode; (B) Negative ion mode**

**Figure S2 Correlation analysis of QC samples: (A) Positive ion mode; (B )Negative ion mode.**
